# The use of behaviour change theory for infection prevention and control practices in healthcare settings: A scoping review

**DOI:** 10.1177/17571774211066779

**Published:** 2022-02-22

**Authors:** Carolynn Greene, Jennie Wilson

**Affiliations:** Richard Wells Research Centre, College of Nursing, Midwifery and Healthcare, 13997 University of West London, London, UK

**Keywords:** behaviour change theory, infection prevention, scoping review

## Abstract

**Background:**

Infection prevention and control (IPC) practices performed by healthcare workers are key to the prevention and management of infections. Compliance with IPC practices is often low, they are therefore commonly the focus of improvement interventions. Designing interventions that are based on behaviour change theories may help to improve compliance to practice. The aim of this review is to synthesise the evidence on the application of behaviour change theories to interventions to improve IPC practice in healthcare settings.

**Methods:**

A scoping review was conducted following the Joanna Briggs Institute methodological framework. The theories of focus were the Theoretical Domains Framework (TDF), Capability, Opportunity, Motivation, Behaviour (COM-B) and Behaviour Change Wheel (BCW). Studies which applied these theories to any IPC practice were included.

**Results:**

Eleven studies were identified which met the inclusion criteria. The IPC behaviours investigated were hand hygiene (7), antimicrobial stewardship (3), and MRSA screening (1). Nine studies explored barriers and facilitators to existing IPC practice; three used their findings to design a behaviour change intervention or tool. Domains of ‘beliefs about consequences’, ‘environmental context/resources’, and ‘social/professional role and identity’ were identified as key across all three IPC behaviours.

**Discussion:**

This review has demonstrated the use of behavioural theories to understand determinants of behaviour related to IPC practice. Currently, there are few published examples of interventions to improve IPC practice that have been underpinned by behavioural theory. Practitioners in IPC should consider the use of these methods to enhance the efficacy of strategies to change healthcare worker behaviour.

## Background

Healthcare associated infections (HCAI) are those which are acquired as a result of receiving treatment in, or visiting, a healthcare setting. Within care settings there are a variety of infection prevention and control (IPC) practices which aim to reduce the occurrence and spread of infection. This includes activities such as hand hygiene, use of personal protective equipment (PPE), screening patients for infection, decontamination of equipment, and antimicrobial stewardship. These practices are supported by a base of research evidence.

Evidence-based guidelines for the prevention of HCAI in acute settings were produced for the National Health Service (NHS) in 2001 (Pratt et al., 2001), and further updated in 2007 and 2014 (Pratt et al., 2007; Loveday et al., 2014). These guidelines were developed through a systematic review of evidence and provide principles of best practice which can be used to inform local procedures in healthcare facilities. The guidelines also provide a standard of practice which can be audited against in order to measure organisational adherence and quality of practice.

Despite many IPC activities being supported by evidence-based guidelines, they are not always complied with by staff. If not implemented effectively then IPC practices risk having little impact. Where practice is poor there may need to be a specific drive on optimising the performance of the IPC behaviour by working with staff to improve practice. Facilitating behaviour change to ensure application of best practice is one of the central roles of the IPC practitioner.

Using behaviour change theory to explore application of evidence-based practices, especially where there may be issues with poor staff performance, allows for the identification of key determinants of behaviour which can be targeted with specific interventions. A variety of behaviour change frameworks and theories have been developed which map the key factors and processes thought to influence behaviour. This tends to incorporate individual factors (e.g. motivations and beliefs), environmental factors (e.g. availability of equipment), as well as complex interactions between individuals and the social and physical contexts they operate within.

There are many different theories of behaviour change, some of which synthesise multiple theoretical components into a single framework. These offer themselves as a practical tool covering a wide scope of factors thought key to behaviour change and can be utilised to inform the development of improvement interventions. The Behaviour Change Wheel (BCW) was developed in 2011 in order to help practitioners from across disciplines to identify appropriate interventions or policies when trying to encourage adoption of a particular behaviour (Michie et al., 2011). The BCW incorporates concepts from 19 existing behaviour change theories and contains the Capability, Opportunity, Motivation, Behaviour (COM-B) model at its centre (Ojo et al., 2019). Capability refers to possessing the psychological or physical capability to perform a behaviour; that is having the knowledge and skills. Opportunity encompasses how the environment, both physical and social, around the individual can prompt behaviour. The motivation to perform behaviour can differ between individuals, this is impacted by both automatic habitual processes and reflective decision making processes. Interaction between these factors influences the performance of behaviour. The BCW also includes potential intervention functions (e.g. education or incentivisation) and policy categories (e.g. regulation or environmental planning) which indicate areas which may drive the required behaviour change.

Each of the COM-B components also map across to another framework, the Theoretical Domains Framework (TDF) (Table 1). Like COM-B the TDF combines aspects of multiple theories, it was developed to support the implementation of evidence-based practice with a focus on changing the behaviour of health professionals (Michie et al., 2005). Through consensus from a group of health psychologists and researchers one hundred constructs derived from 33 behaviour change theories were reduced into a framework of 12 domains, each thought to play a key role in behaviour change with a focus on clinical practice. The TDF was later validated for use in implementation research, at this time two domains were added giving a total of 14 domains in the framework (Cane et al., 2012). As COM-B and TDF share similar constructs they can be used in conjunction. For instance, Michie et al. (2014) suggest the COM-B can be used to identify relevant components to the behaviour of interest, from these the relevant TDF domains can be identified and used to further explore and interrogate these factors more deeply.

**Table 1.** Overview and definition of domains from COM-B and TDF (Adapted from Cane et al., 2012; Michie et al., 2014).

COM-B component	Theoretical domains framework (TDF) domain	Definitions
Capability (psychological or physical)	Knowledge	An awareness of the existence of something
	Behavioural regulation	Anything aimed at managing or changing objectively observed or measured actions
	Memory, attention and decision processes	The ability to retain information, focus selectively on aspects of the environment, and choose between 2 or more alternatives
	Skills	An ability or proficiency acquired through practice
Opportunity (social or physical)	Social influences	Those interpersonal processes that can cause individuals to change their thoughts, feelings, or behaviours
	Environmental context and resources	Any circumstance of a person’s situation or environment that discourages or encourages the development of skills and abilities, independence, social competence, and adaptive behaviour
Motivation (automatic or reflective)	Beliefs about capabilities	Acceptance of the truth, reality, or validity about an ability, talent, or facility that a person can put to constructive use
	Beliefs about consequences	Acceptance of the truth, reality, or validity about outcomes of a behaviour in a given situation
	Emotions	A complex reaction pattern involving experiential, behavioural, and physiological elements, by which the individual attempts to deal with a personally significant matter or event
	Goals	Mental representations of outcomes or end states that an individual wants to achieve
	Intentions	A conscious decision to perform a behaviour or a resolve to act in a certain way
	Optimism	The confidence that things will happen for the best or that desired goals will be attained
	Reinforcement	Increasing the probability of a response by arranging a dependent relationship, or contingency, between the response and a given stimulus
	Social/Professional role and identity	A coherent set of behaviours and displayed personal qualities of an individual in a social or work setting

The BCW and TDF combine and simplify several different behaviour change theories in order to create a tool or framework which can be used by practitioners across various disciplines (Cane et al., 2012). Designing and facilitating behaviour change interventions for IPC practices is a key part of the IPC practitioner’s role. Utilisation of theory to underpin interventions provides a sound theoretical base which may have an important impact on outcomes. There is some evidence that interventions which are underpinned by theory are more likely to be effective (Michie and Johnston, 2012).

The purpose of this review is to explore how behaviour change theory has been applied to IPC practices in healthcare settings, to identify common themes and consider any implications for practice. Due to their focus on clinical practice the theories of interest are the TDF, COM-B and the BCW.

## Methods

In order to explore the existing literature a scoping review was conducted. Scoping reviews are a way of systematically mapping an area of research evidence and generate a descriptive overview exploring the extent, range, and characteristics of published evidence for a particular topic (Pham et al., 2014). This highlights the types of evidence available and gaps in the existing literature. As scoping reviews aim to provide a wide overview of existing studies in a particular area a formal quality assessment is not relevant (Peters et al., 2015). The objective of this scoping review was to explore how behaviour change theories have been applied to IPC practices in healthcare settings. The review followed the Joanna Briggs Institute (JBI) methodology for conducting scoping reviews. The main review question was: How have behaviour change theories been applied to IPC practices in healthcare settings?

### Inclusion/exclusion criteria

Evidence from primary research, both quantitative and qualitative, was included to ensure a broad range of studies were located. This kept the scope wide and ensured the map of the literature was thorough. Grey literature was not searched. Relevant theories were TDF, BCW or COM-B and any type of IPC practice was included. The review focused on literature relating to healthcare settings, including care homes. Only studies published in the English language and published since the year 2000 were included as the relevant behaviour change theories were developed following this date. Text, review, opinion papers and letters were excluded.

### Search strategy

A three-step strategy as recommended by the JBI was undertaken. This comprised (1) an initial search undertaken to identify relevant keywords and search terms. This informed (2) an individual search strategy developed for each database including mapping to relevant subject headings. Databases searched were CINAHL Complete, EMBASE and MEDLINE. Lastly, (3) the reference lists of relevant papers were screened to identify any additional studies. A search strategy for MEDLINE is detailed in Appendix 1. Limits applied to the search were: papers published in the English language, published after the year 2000, and with an abstract available.

Two reviewers assessed all titles for relevance. Relevant papers were retrieved and resifted by both reviewers. Disagreements were resolved through discussion. Information on authors, country of origin, publication year, type of theory used, methods, and key findings were extracted into a charting table (Supplementary Material 1), a brief charting table is also included (Table 2) and the findings incorporated into a narrative summary.

**Table 2.** Brief charting table of reviewed papers.

Authors and country of publication	Relevant theory	Overview of study
Hand hygiene (HH)		
1) Boscart et al. (2012), Canada	TDF	Identified nurses and administrators perceived barriers and facilitators to HH practices and introduction of an electronic monitoring system for HH.
2) Dyson et al. (2011), UK	TDF	Exploration of a theory-informed and non–theory-informed question schedule to assess barriers and levers to HH.
3) Dyson et al. (2013), UK	TDF	Development of an instrument to measure barriers and levers to HH.
4) Fuller et al. (2014), UK	TDF	An exploration of real-time explanations of HH noncompliance
5) McAteer et al. (2014), UK	TDF	Exploration of barriers and facilitators to implementation of HH intervention by those who delivered it
6) Smith et al. (2019), Canada	TDF	Exploration of barriers and facilitators to HH in long-term care facilities through development of a theory-informed questionnaire
7) Squires et al. (2014), Canada	TDF	Exploration of the barriers and facilitators to physician HH compliance
Antimicrobial stewardship (AMS)		
8) Chambers et al. (2019), Canada	TDF	Exploration of barriers and facilitators that contribute to overuse of antibiotics for urinary tract infection (UTI) in long-term care. Developed a theory-informed AMS programme
9) Fisher et al. (2018), Canada	TDF and COM-B	Determination of the barriers and facilitators to promotion of intravenous to oral antimicrobial stepdown by nurses
10) Jones et al. (2018), UK	TDF and COM-B	Investigation of the attitudes towards and experiences of AMS for community pharmacies in order to explore barriers and opportunities to AMS.
MRSA screening		
11) Currie et al. (2019), UK	TDF	Identification of factors which influenced staff compliance with MRSA screening policies

## Results

**Figure 1.**
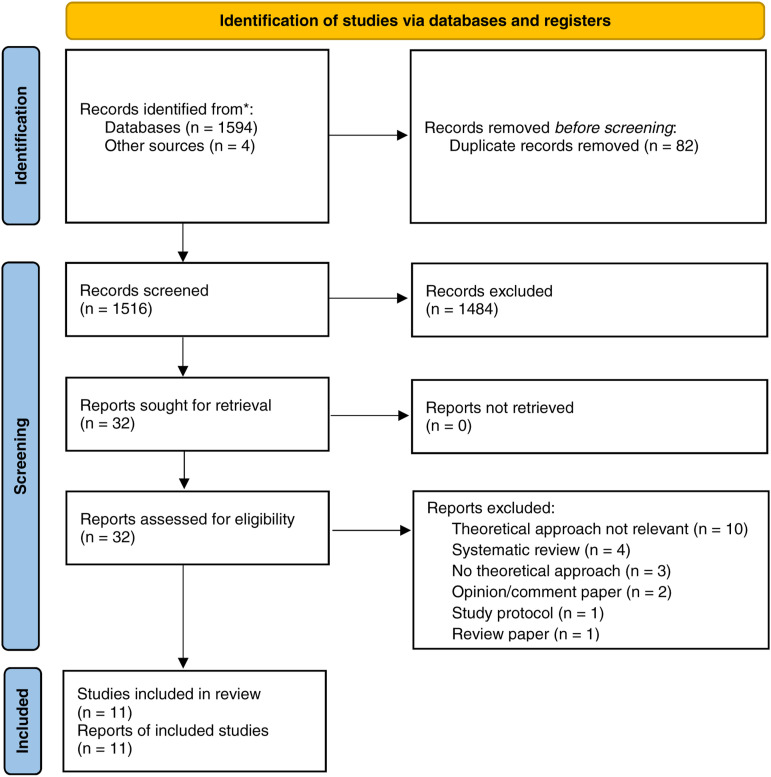
PRISMA flow diagram for the scoping review process (Adapted from Page et al., 2021).

The scoping review identified 1516 papers after removal of duplicates, of which 11 were relevant to the research question and included in the review. A PRISMA diagram is presented in Figure 1 (Page et al., 2021). These papers were published between the years 2011 - 2019 and were from two countries: United Kingdom (*n* = 6) and Canada (*n* = 5). Results are organised by the type of IPC practice the study focused upon. Of the 11 included studies seven focused on hand hygiene, three on antimicrobial stewardship, and one on methicillin-resistant *Staphylococcus aureus* (MRSA) screening (Table 2).

### Hand hygiene

Hand hygiene is a key behaviour in the interruption of the spread of pathogens during patient care. Performance of hand hygiene by healthcare workers at specific moments during care provision aims to reduce the spread of infection between sites on one patient, between different patients, and around the healthcare environment. Seven papers were identified which had used the relevant theories to explore staff performance of hand hygiene in healthcare environments. The studies focused on three different aspects of hand hygiene: barriers and facilitators, decision making, and intervention success.

### Barriers and facilitators to hand hygiene

Five studies explored the barriers and facilitators to appropriate hand hygiene behaviour. Settings included long-term care (Smith et al., 2019) and hospital (Dyson et al., 2011, 2013; Boscart et al., 2012; Squires et al., 2014). All five studies included interviews or questionnaires about the performance of hand hygiene with frontline staff as participants. All used the TDF to inform the development of question schedules and the analysis and interpretation of data.

Smith et al. (2019) aimed to identify key attitudes and barriers and facilitators to hand hygiene in the care home setting by designing a staff survey. An initial survey of 85 care workers narrowed the questions, which were based on existing surveys and literature and mapped on to the TDF domains. From this, a second survey was developed which contained 47 closed-ended questions. Analysis of the second survey focused on 342 staff whose role included providing direct care. This survey identified four main themes which mapped on to three TDF domains. The barriers to hand hygiene were related to the domain of environmental context and resources, this included time pressure, workload, and environmental controls. Hand hygiene was facilitated by two domains, that of social/professional role and identity, and beliefs about consequences to self and others. This encompassed performance of hand hygiene feeling like part of their professional duty, and its potential impact on themselves, co-workers, and patients. The authors noted that the barriers they identified were similar to those seen in hospitals. Smith et al. (2019) saw the resulting questionnaire as a useful tool for defining key factors which may restrict or encourage hand hygiene behaviour in an organisation which can then inform selection of appropriate interventions specific to that setting.

As compliance to hand hygiene may differ depending on job role, theories can support the exploration of potential determinates of behaviour for these different groups. Looking at specific job roles, Squires et al. (2014) interviewed 42 physicians, both staff and residents from surgical and medical wards, using a question schedule informed by the TDF. Nine of the 14 domains from the TDF were identified as relevant to hand hygiene practice: (i) knowledge, (ii) skills, (iii) beliefs about capabilities, (iv) beliefs about consequences, (v) goals, (vi) memory, attention, and decision processes, (vii) environmental context and resources, (viii) social professional role and identity; and (ix) social influences. The authors found that physicians reported a knowledge and skills gap related to guidelines and performance of hand hygiene. This was surprising given it would be expected these areas would be covered during training. The influence of just one domain, social influences, differed between the specialities with more surgical staff reporting their team influenced their performance of hand hygiene than the medical staff. It was noted that nearly all participants thought performance of hand hygiene was a conscious process, thus may benefit from reminders in the environment.

Dyson et al. (2011) explored barriers and facilitators to hand hygiene whilst comparing data elicited from two types of questionnaire. One questionnaire was developed using the TDF, with questions covering 12 domains. The other questionnaire was based on existing literature and probed existing factors which have been found to influence hand hygiene including social and organisational; individual differences; and knowledge. Questions were delivered via focus groups, interviews, and paper questionnaires with a total of 70 healthcare workers. The authors found that the theory-based questions prompted significantly more discussion of three domains in particular: emotion, habit/routine, and incentives. The authors suggest that these domains may have an unconscious influence upon behaviour, thus by asking participants about them outright their influence is considered and discussed.

The TDF has also been used to underpin the design of an instrument to explore barriers and facilitators to hand hygiene which can be administered to large groups. Through use of Delphi survey and pilot testing Dyson et al. (2013) developed an instrument which consisted of 33 questions spanning 10 TDF domains. Testing with healthcare workers showed that those who reported higher numbers of barriers had lower self-reported compliance to hand hygiene. The authors propose that development of such instruments allows for large scale assessment of healthcare staff in an organisation as opposed to potentially lengthy interview processes. This also allows for tailored interventions to be developed based on local results.

As theory can help to explore influences on performance of evidence-based practice it can be used to explore potential barriers to practice before interventions are implemented. Boscart et al. (2012) aimed to explore barriers and facilitators to the introduction of a new electronic monitoring system (EMS) for hand hygiene as well as to existing hand hygiene practice in a hospital setting. 10 interviews, with questions informed by the TDF, were conducted with nursing staff and administrative staff (IPC nurse, unit manager, and director of care). The authors found differences between the responses from the nurses and administrators. In general, nurses felt they had sufficient knowledge, skills and capabilities to perform hand hygiene, and discussed the routine nature of hand hygiene to their practice. Administrators thought nurses potentially lacked in knowledge and decision making and identified potential environmental barriers nurses may encounter to performance of hand hygiene. Discussing hand hygiene practice and the EMS enabled the authors to pinpoint specific areas they could target when implementing the EMS in order to aid its success. Interviews also highlighted differences in views dependent on job role which could be considered when planning implementation strategies.

### Staff decision making

The decision for individuals to perform hand hygiene is influenced by both automatic and conscious processes. To explore how healthcare staff decided when to clean their hands during practice Fuller et al. (2014) observed care provision and asked staff about their noncompliance to hand hygiene immediately following the event. The TDF was used to code and analyse the reasons given for noncompliance. Just over two thirds (142/207, 67%) of coding related to two domains of the TDF; (1) memory, attention and decision processes and (2) knowledge. Fuller et al. (2014) surmised that this indicated that both automatic and conscious process need to be targeted when designing interventions due to the dynamic nature of behavioural influences.

### Interventions to improve hand hygiene

Hand hygiene is often the focus of improvement interventions. McAteer et al. (2014) explored why an intervention may succeed in some settings but not others. They assessed the implementation of an intervention to improve hand hygiene which was trialled using a stepped wedge cluster randomised controlled trial in 16 NHS trusts. The intervention itself was based on goal setting and control theory, involving observation of staff, feedback and goal setting. Ward coordinators, who delivered the intervention, from 17/33 (52%) of the wards involved were interviewed to explore experienced successes and challenges. Interview questions were based on nine TDF domains thought most relevant to the topic, answers related to these domains were coded with a number which represented how likely it was to contribute to intervention success. McAteer et al. (2014) found that domains most related to successful implementation were linked to professional identity in that the tasks were already part of the ward coordinator role, knowledge of the intervention, skills around implementation, motivation to deliver the intervention, and behavioural regulation with regard to prioritising goals.

### Antimicrobial stewardship

Antimicrobial stewardship focuses on optimising the use of antibiotics in order to minimise unnecessary use, or overuse. This is considered to be critical in reducing and controlling the emergence of antimicrobial resistant pathogens. Three studies were found which focused on perceived barriers and facilitators to antimicrobial stewardship. All were based in different settings covering long-term care (Chambers et al., 2019), hospital (Fisher et al., 2018) and community pharmacy (Jones et al., 2018). In all studies, the TDF was used to inform a question schedule or analyse data collected via interviews or surveys. Two studies (Fisher et al., 2018; Jones et al., 2018) went on to map the identified domains onto the COM-B to ascertain the relevant behaviour change techniques.

Fisher et al. (2018) used semi-structured interviews with 15 nurses at one hospital to explore the barriers and facilitators to stepdown from intravenous (IV) to oral antibiotics on hospital wards. Interview schedules were developed using the TDF and responses analysed using content analysis focusing on the TDF domains. All TDF domains, except that of emotion were represented in the data. More than half of the coded responses represented just four domains: beliefs about consequences, knowledge, environmental context and resources, and social/professional role and identity. Domains were mapped onto the COM-B system in order to identify the potential development of interventions to promote the stepdown to oral antibiotics.

Jones et al. (2018) focused on current and potential use of antimicrobial stewardship in the community pharmacy setting. This was explored through interviews and focus groups with 58 participants working within community pharmacies and GP surgeries. The question schedule was informed by the TDF, with responses showing comments coded into all 14 domains. Identified domains were mapped onto COM-B to identify relevant interventions, and this led to recommendations as to how practice could be improved. Recommendations were focused on four key TDF domains: environmental context and resources, beliefs about consequences, memory, attention and decision making, and professional role and identity.

One study developed a theory-informed antimicrobial stewardship programme. Chambers et al. (2019) explored the barriers and facilitators to management and treatment of urinary tract infections by surveying 381 people working in long-term care. Responses were coded and mapped onto the TDF domains; this identified eight domains as relevant to appropriate prescribing practice. Relevant domains were then mapped onto a specialised database which suggested interventions to improve drug prescribing practice. Interventions were chosen which had the potential to address the TDF constructs identified. Focus groups with staff from two long-term care facilities were held to explore acceptability and feasibility of proposed interventions in the care setting.

### MRSA screening

The routine screening of patients for MRSA helps to appropriately manage those colonised and reduce the risk to other patients. One study used a mixed methods approach to explore the MRSA screening behaviours of UK hospital staff (Currie et al., 2019). The TDF was used to design a question schedule and analyse the results of interviews and focus groups with 49 nurses and clinical staff. This identified key barriers and enablers to screening behaviour which were used to design a national survey to explore the issue. Three-quarters of survey respondents (76%, 343/450) reported their compliance with MRSA screening procedures as >90%, this was considered optimum compliance according to local standards. Logistic regression found three predictors for >90% compliance: (1) screening as part of admission process (it was seen as easy to complete due to admission routine); (2) feedback regarding compliance levels to screening (staff were aware of their performance); and (3) clinical area (the influence of ward culture). The authors recommend targeting these areas in order to influence and embed screening behaviour.

## Discussion

This scoping review has shown how behaviour change theories have been used to explore the application of evidence-based IPC practices in relation to hand hygiene, antimicrobial stewardship, and MRSA screening. The reviewed studies encompassed a range of settings and staff roles with most exploring perceived barriers and facilitators to existing IPC practices by healthcare staff. This can help to explore determinants of engrained practice and identify potential interventions specific to the setting. Only three of the studies (Dyson et al., 2013; Chambers et al., 2019; Smith et al., 2019) described an intervention or development of a tool which targeted the behavioural determinants identified.

Use of a theoretical framework within the studies ensured a wide range of behavioural determinants were explored, including ones which were not previously reported to be of influence on the particular behaviour. This is demonstrated by Dyson et al.’s (2011) finding that theory-informed questions elicited discussion from participants of a wider scope of behavioural determinants than questions based on published literature. This broader assessment of the range of barriers and facilitators identifies potentially unknown influences on IPC behaviours which can be targeted in the design of interventions.

Some domains were frequently identified across all three IPC behaviours: beliefs about consequences, environmental context and resources, and social/professional role and identity (Table 3). These may be key areas to consider when planning interventions in IPC practice. Awareness of the consequences of an infection occurring or its potential spread to other patients or the healthcare worker themselves was a facilitator for performance of hand hygiene (Smith et al., 2019). However, attribution of the occurrence of infection to behaviour is problematic due to the period of intermission between the two events. Due to this delay in consequences, encouraging IPC behaviour may require greater focus on the formation of habitual behaviour and developing emotion-based motivations to perform behaviour (Cioffi and Cioffi, 2014). Where a specific behaviour was to be avoided, e.g. prescribing antimicrobial agents, some healthcare workers worried that if a patient was not treated they may develop an infection (Chambers et al., 2019). This perception of a potentially negative consequence can be addressed by providing support and education to promote recognition of the balance between the appropriate use of antibiotics and potential harm from over usage.

**Table 3.** The TDF domains identified in reviewed studies*.

	Domains identified in each study		
Theoretical domains framework (TDF) domain	Hand hygiene	Antimicrobial stewardship	MRSA screening
Behavioural regulation	1, 2, 3, 4, 5, 6	9, 10	11
Beliefs about capabilities	1, 3, 5, 6, 7	9, 10	—
Beliefs about consequences	1, 2, 3, 4, 6, 7	8, 9, 10	11
Emotions	1, 2, 3, 4, 5	8, 10	—
Environmental context and resources	1, 2, 3, 4, 5, 6, 7	8, 9, 10	11
Goals	1, 7	9, 10	—
Intentions	1	9, 10	—
Knowledge	1, 2, 3, 4, 5, 7	8, 9, 10	—
Memory, attention and decision processes	1, 2, 3, 4, 6, 7	9, 10	—
Optimism	1	9, 10	—
Reinforcement	1, 2	8, 10	11
Skills	1, 2, 3, 4, 5, 7	8, 9, 10	—
Social influences	1, 2, 3, 4, 5, 6, 7	8, 9, 10	—
Social/Professional role and identity	1, 2, 3, 5, 6, 7	8, 9, 10	11

*Studies identified by numbers used in Table 2.

In order for staff to adhere to preferred IPC behaviours they require an environment that supports these actions. For hand hygiene the location of alcohol-based hand rub at the point-of-care enables healthcare workers to decontaminate their hands close to where contamination occurs. This point-of-care location has been found to increase compliance to hand hygiene (Traore et al., 2007). This sort of environmental or resource change may require the introduction of new systems and processes or adapting something which is already in place. The idea of an enabling environment also links to the concept of making IPC behaviours an essential part of the professional role. Creating an environment which encourages IPC behaviours makes it easier for them to be performed as a core part of everyday practice and create a strong link to a sense of professionalism.

Other domains featured in some studies but not in others, although sometimes this was due to questionnaire design and whether they included all domains in the questions. The differences between the findings of the studies also demonstrates the importance of exploring determinants of behaviour within individual settings rather than assuming we understand why a behaviour is, or is not, performed consistently. Identification of these specific barriers and facilitators is vital before designing or introducing interventions. Engaging staff in this process may also demonstrate to them that any intervention to be introduced will consider issues specific to their experience and context.

The influence of different factors on behaviour was shown to vary according to occupational group (Boscart et al., 2012; Dyson et al., 2013; Squires et al., 2014). This is of importance when thinking about improving IPC practice in a ward setting where different team members may benefit from tailored support or different approaches to training. Squires et al. (2014) found a lack of knowledge and skills around hand hygiene among physicians even though it would be part of their basic training. Assumptions may commonly be made about level of knowledge and skills in relation to IPC practices, therefore additional ward-based training and feedback may benefit staff (Tavolacci et al., 2008).

Most studies in this review involved interviews with staff, these can be time consuming to complete and analyse. The survey instrument developed by Dyson et al. (2013) demonstrates an approach for assessing determinants of behaviour at scale across an organisation, obviating the need for interviews. By developing such theory-based instruments, IPC practitioners can target larger cohorts of staff across different settings to define the specific factors influencing behaviour at a local level. Ensuring these tools have an underlying theoretical base also allows for relevant behaviour change techniques to be identified and included in the design of interventions.

The successful implementation of behaviour change strategies is key to the effectiveness of interventions. Using behaviour change theory to explore potential barriers and facilitators prior to the design, or implementation, of an intervention allows for it to be tailored to each specific context. In addition, after an intervention has been implemented the reasons behind its success or failure can be explored using the same framework (McAteer et al., 2014). This can highlight key areas to address or support when implementing interventions in similar settings, or to inform adaptions to improve the intervention.

### Limitations

The scope of the identified studies is currently fairly narrow and focused on exploring three IPC practices. Some papers relied on self-reported compliance to IPC behaviour, this could have led to social desirability bias where participants report they perform behaviour more than they do in reality. Combining staff interviews with observation of care delivery or reviewing audit data may present a more accurate picture of compliance where this is important. The studies identified for inclusion in this review were only conducted in two countries, the UK and Canada, thus behavioural determinants may vary further depending on the country the research is conducted in. Future research should aim to extend the scope of theory-based analysis of behaviour related to a wider range of IPC practices. Areas of interest could include use of PPE including glove use, implementation of care pathways and bundles, and adherence to isolation precautions.

## Conclusions

Use of behaviour change theories to explore IPC practices has helped to establish a range of determinants involved in the performance of behaviour. Identifying these factors means they can be targeted in order to support the translation of evidence into practice, ensuring it meets recommended standards and guidelines. It would be of benefit for IPC practitioners to utilise these methods to explore practice and support behaviour change. The small number of published studies and IPC behaviours explored indicate more research in this area is required which is underpinned by theoretical frameworks.

## Supplemental Material

sj-pdf-1-bji-10.1177_17571774211066779 – Supplemental Material for The use of behaviour change theory for infection prevention and control practices in healthcare settings: A scoping review Click here for additional data file. Supplemental Material, sj-pdf-1-bji-10.1177_17571774211066779 for The use of behaviour change theory for infection prevention and control practices in healthcare settings: A scoping review by Carolynn Greene, and Jennie Wilson in Journal of Infection Prevention

## Appendix 1 Search strategy for MEDLINE

Exp Models, Theoretical/OR exp Psychological Theory/OR Theoretical Domains Framework.mp. OR COM-B.mp. OR Behaviour change wheel.mp. AND exp Antimicrobial Stewardship/OR exp Hand Disinfection/OR exp Disease Transmission, Infectious/pc (Prevention & Control) OR infection prevention.mp. OR exp Cross Infection/pc (Prevention & Control) AND exp “Quality of Health Care"/OR exp Program Development/OR improvement.mp Limit to (abstracts and English language and yr="2000 -Current").
