# Supplementary material for: The use of behaviour change theory for infection prevention and control practices in healthcare settings: A scoping review
Source: J Infect Prev. 2022 Feb 22;23(3):108–17. doi: 10.1177/17571774211066779 (PMC9052851; doi:10.1177/17571774211066779)
Supplement: sj-pdf-1-bji-10.1177_17571774211066779 – Supplemental Material for The use of behaviour change theory for infection prevention and control practices in healthcare settings: A scoping review [file sj-pdf-1-bji-10.1177_17571774211066779.pdf]

Supplementary Material 1: Charting table

Author(s) and year	Country of Origin	Aims/Purpose	Participants	Setting	Methods (inc. theory used)	Findings	Outcomes	Key findings that relate to review question (inc. limitations)
Boscart, V. M. et al. (2012) 'Using psychological theory to inform methods to optimize the implementation of a hand hygiene intervention'	Canada	Identify nurses' and administrators perceived barriers and facilitators to HH practices and introduction of an electronic monitoring system (EMS) for HH.	10 (7 nurses, 3 administrators: 1 unit manager, 1 director of care, 1 IPC nurse).	One 50-bed hospital unit.	Qualitative in-depth semi-structured interviews.<br><br>Theory: TDF to develop questions related to domains.	All TDF domains included in responses. Responses differed between nurses and administrative staff for most of the domains.	Identification of barriers and facilitators to HH. Findings informed how implementation of an EMS was designed.	TDF used to explore barriers and facilitators to HH. Informed planning intervention implementation with use of behaviour change techniques and feedback.<br><br>Limitations: social desirability bias, simple coding scheme, small sample from one hospital.
Chambers, A. et al. (2019) 'A recipe for antimicrobial stewardship success: Using intervention mapping to develop a program to reduce antibiotic overuse in long-term care'	Canada	To explore barriers and facilitators that contribute to overuse of antibiotics for UTI in long-term care (LTC). To use this information to inform an evidence and theory-informed antimicrobial stewardship program.	Survey: 381/643 (59% response rate) staff working in LTC, including administrators, directors, Registered Nurses, physicians, IPC practitioners.	Long term care facilities.	Intervention mapping approach using cross-sectional survey and focus groups.<br><br>Theory: TDF.	19 barriers and facilitators mapped onto 8 TDF domains: knowledge, skills, environmental context and resources, social or professional role or identity, social influences, beliefs about consequences, emotions, and reinforcement. These informed the selection of nine strategies to support	Assessment of barriers and facilitators lead to the design of a multifaceted approach for implementation of an antimicrobial stewardship program.	Use of TDF to identify behaviour change meant relevant implementation strategies could be pinpointed.<br><br>Limitations: Did not seek opinions from LTC residents or relatives. Barriers and facilitators may be specific to the participants involved.

						implementation of the antimicrobial stewardship program.		
Currie, K. et al. (2019) 'Barriers and enablers to meticillin-resistant Staphylococcus aureus admission screening in hospitals: a mixed-methods study'	UK	Identify factors which influence staff compliance with MRSA screening policies.	Stage 1: Qualitative interviews (telephone and focus groups); 7 focus groups, 38 staff in total. Individual interviews, 11 participants.<br><br>Stage 2: Cross-sectional national survey: 478/558 (86%) response rate; 450 analysed.	Stage 1: Four different Health Board sites; NHS clinical staff.<br><br>Stage 2: National survey of 15 Health Boards in Scotland.	Sequential two-stage mixed-methods.<br><br>Theory: TDF.	Stage 1: two key themes; a) the role of culture and b) immediate environment and hospital procedures.<br><br>Stage 2: 76.2% of respondents met target (>90%) of screening for MRSA. Three significant predictors of compliance; 1) routinized nature of MRSA screening within admission process; 2) provision of information relating to MRSA compliance within the recipient's clinical area; and 3) category of 'clinical area'.<br><br>Five TDF domains were identified as key: behavioural	Recommendations made for implementing and embedding MRSA screening.	Use of theoretical frameworks provided depth of understanding the mechanisms of implementations for future interventions.<br><br>Limitations: adherence to MRSA screening was self-report by staff and not verified.

						regulation, beliefs about consequences, environmental context and resources, skills, social/professional role and identity.		
Dyson, J. et al. (2013) 'Development of a theory-based instrument to identify barriers and levers to best hand hygiene practice among healthcare practitioners'	UK	To explore barriers to performance of evidence-based practice (hand hygiene), by way of developing and instrument informed by TDF.	Delphi survey: two rounds, 21 IPC experts.<br><br>Pilot study: 10 participants.<br><br>Instrument testing: a) 56/150 questionnaire responses, b) 354/900 responses, c) 50/150 responses.	Three UK hospitals.	Delphi survey.<br><br>Theory: TDF.	Starting with 100 barriers and levers to hand hygiene the study produced an instrument with 33 items across 10 TDF domains. Then tested for validity and reliability.<br><br>Barriers and facilitators for performance of HH varied across occupational role.	Development of the Barriers and Levers to Hand Hygiene Instrument (BALHHI).	As developed using the TDF the instrument can identify appropriate behaviour change strategies to target hand hygiene behaviour.
Dyson, J. et al. (2011) 'Does the use of a theoretical approach tell us more about hand hygiene behaviour? the barriers and levers to hand hygiene'	UK	To evaluate the use of psychological theory in assessing barriers and levers to hand hygiene.	70 healthcare practitioners.<br><br>Interviews (25 participants), Focus groups (3 groups, 21 participants), Questionnaire (24/65 respondents)	Three UK NHS trusts.	Survey.<br><br>Theory: TDF.	Two questionnaires used: one informed by TDF, one informed by existing literature.<br><br>The theory informed questionnaire resulted in more participants	The theory-based questionnaire identified further barriers and levers to HH.	Theory-based questionnaire elicited non-conscious influences on HH which we may be unaware of.<br><br>Limitations: possible social desirability bias; each questionnaire was not equally distributed across job role.

						discussing the influence of TDF domains: emotion, routine/habit, and incentives.		
Fisher, C. C. et al. (2018) 'A theory-informed assessment of the barriers and facilitators to nurse-driven antimicrobial stewardship'	Canada	Determine barriers and facilitators to promotion of intravenous (IV) to oral (PO) antimicrobials step-down by nurses.	Semi-structured individual interviews with 15 nurses (8 Registered Nurses and 7 Licensed Practical Nurses).	One 400-bed hospital, three wards.	Prospective qualitative descriptive study.<br><br>Theory: TDF, mapped on to COM-B.	All domains in TDF were reflected in the interview responses except that of emotion.<br><br>The most frequently occurring responses fell into four domains: beliefs about consequences, knowledge, environmental context and resources, and social and professional role and identity.	Identification of barriers and facilitators highlighted areas which future behaviour change interventions should target.	Use of TDF and COM-B meant relevant domains and potential interventions were identified.<br><br>Limitation: small sample size, individual interviews only; focus groups may have facilitated further discussion.
Fuller, C. et al. (2014) 'Application of a theoretical framework for behavior change to hospital workers' real-time explanations for	UK	To explore 'real-time' explanations of HH noncompliance.	Healthcare workers.<br><br>570 observer feedback forms completed; 209 showing non-compliance with 185 documenting reasons for non-	13 English and Welsh hospitals.	Cross-sectional study within a cluster randomised control trial (Feedback Intervention Trial).<br><br>Theory: TDF.	Observers asked HCW to explain their non-compliance, these were documented and coded using the TDF.<br><br>Just over 2/3 of 207 explanations were coded into two domains: 1)	Interventions may need to target automatic processes rather than assuming HH behaviour is a conscious action.	Use of TDF covered more areas than a single behaviour theory.<br><br>Limitations: Social desirability bias as observers were senior ward staff. Explanations for noncompliance were not recorded verbatim, some detail may have been lost.

noncompliance with hand hygiene guidelines’			compliance.			memory, attention, and decision processes (42%) and 2) knowledge (26%).		
Jones, L. F. et al. (2018) ‘Qualitative study using interviews and focus groups to explore the current and potential for antimicrobial stewardship in community pharmacy informed by the Theoretical Domains Framework’	UK	Investigate attitudes and experiences of antimicrobial stewardship (AMS) for community pharmacies in order to explore barriers and opportunities to AMS.	Interviews and focus groups with 58 participants. 8 GPs, 28 pharmacists, 13 pharmacy staff, 6 reps from pharmacy organisations, and 2 local stakeholders.	Stratified group of pharmacies in Birmingham, Gloucestershire and South Wales. 25/120 (21%) contacted participated.	Qualitative study; interviews and focus groups<br><br>Theory: TDF and COM-B.	Interview responses were coded into all TDF domains. Key domains recommended for intervention development using COM-B were: 1) Environmental context and resources 2) Beliefs about consequences 3) Memory, attention and decision-making 4) Professional role and identity.	Findings were used to develop recommendations for interventions along with suitable behaviour change techniques.	Use of theory allowed for development of question schedule, interpretation of findings and intervention recommendation.
McAteer, J. et al. (2014) ‘Using psychological theory to understand the challenges facing staff delivering a ward-led intervention to increase hand	UK	Explore barriers and facilitators to implementation of HH intervention by those delivering intervention.	17/33 participants – ward coordinators who implemented an intervention program.	Participants from 11 hospitals.	Qualitative study within a cluster randomised control trial (Feedback Intervention Trial).<br><br>Theory: TDF.	Each domain was given a rating representing contribution to intervention success; ‘good’ (1), ‘partial’ (.5) or ‘no evidence’ (0).<br><br>Highest scoring (>12.5): behavioural	Focusing on the domains which were key to success could maximise the impact of the intervention.	Investigates theoretical explanations for successful implementation of a HH intervention.

hygiene behavior: A qualitative study’						regulation, motivation, skills, knowledge, social or professional role and identity. Lowest scoring (<8.5): environmental context and resources, beliefs about capabilities, social influence, and emotion.		
Smith, J. D. et al. (2019) ‘Application of the Theoretical Domains Framework to identify factors that influence hand hygiene compliance in long-term care’	Canada	To explore barriers and facilitators to HH in long-term care facilities using a theory informed questionnaire.	Phase 1: 10 HCWs from 10 LTC facilities with 85/100 responses.<br><br>Phase 2: 10 HCWs from 70 LTC facilities with 420/700 responses, 342 of whom provided direct care.	Staff from LTC facilities.	Cross-sectional mixed methods survey.<br><br>Theory: TDF.	Phase 1: questionnaire of 30 closed-ended questions and 3 open-ended. Content analysis and factor analysis informed the development of the questionnaire for Phase 2.<br><br>Phase 2: questionnaire of 47 closed-ended questions exploring potential barriers and facilitators. Resulting model contained four themes related to TDF domains: (i) social/professional	Development of a theory-informed survey which can identify domains of the TDF which can be used to target interventions to improve HH compliance.	Use of TDF allowed for identification of influences on behaviour which may not have been considered.

						role and identity; (ii) resource barriers related to time pressure and workload, (iii) beliefs about consequences to self and others, and (iv) resource barriers related to environmental controls. Barriers were similar to those in acute care.		
Squires, J. E. et al. (2014) 'Understanding Practice: Factors That Influence Physician Hand Hygiene Compliance'	Canada	To explore the barriers and facilitators to physician HH compliance using questions informed by the TDF.	42 staff physicians and residents (internal medicine and surgery).	One 1100-bed multisite tertiary care hospital.	Descriptive qualitative study using thematic content analysis.<br><br>Theory: TDF.	Nine of the 14 domains from the TDF were identified as relevant. There were no differences in relevant domains between specialty or role except 1) social influences which was impacted by specialty; more surgical staff reported their hand hygiene behaviour was influenced by their team compared to medical staff.	Use of TDF identified targets for behaviour change interventions. Knowledge was a key area which requires focus and may currently be overlooked.	Identified targets for behaviour change interventions, due to the number of relevant domains this would be expected to be a multifaceted intervention.

[HCWs = healthcare workers, HH = hand hygiene, LTC = long-term care, TDF = Theoretical Domains Framework]
